# Axonal transport and secretion of fibrillar forms of α-synuclein, Aβ42 peptide and HTTExon 1

**DOI:** 10.1007/s00401-016-1538-0

**Published:** 2016-01-28

**Authors:** Michel Brahic, Luc Bousset, Gregor Bieri, Ronald Melki, Aaron D. Gitler

**Affiliations:** Department of Genetics, Stanford University School of Medicine, Stanford, CA 94305-5120 USA; Paris-Saclay Institute of Neuroscience, CNRS, Gif-sur-Yvette, France; Neurosciences Graduate Program, Stanford University School of Medicine, Stanford, CA USA

**Keywords:** α-Synuclein, Aβ42, HTTExon1, Axonal transport, Secretion

## Abstract

Accruing evidence suggests that prion-like behavior of fibrillar forms of α-synuclein, β-amyloid peptide and mutant huntingtin are responsible for the spread of the lesions that characterize Parkinson disease, Alzheimer disease and Huntington disease, respectively. It is unknown whether these distinct protein assemblies are transported within and between neurons by similar or distinct mechanisms. It is also unclear if neuronal death or injury is required for neuron-to-neuron transfer. To address these questions, we used mouse primary cortical neurons grown in microfluidic devices to measure the amounts of α-synuclein, Aβ42 and HTTExon1 fibrils transported by axons in both directions (anterograde and retrograde), as well as to examine the mechanism of their release from axons after anterograde transport. We observed that the three fibrils were transported in both anterograde and retrograde directions but with strikingly different efficiencies. The amount of Aβ42 fibrils transported was ten times higher than that of the other two fibrils. HTTExon1 was efficiently transported in the retrograde direction but only marginally in the anterograde direction. Finally, using neurons from two distinct mutant mouse strains whose axons are highly resistant to neurodegeneration (Wld^S^ and Sarm1^−/−^), we found that the three different fibrils were secreted by axons after anterograde transport, in the absence of axonal lysis, indicating that trans-neuronal spread can occur in intact healthy neurons. In summary, fibrils of α-synuclein, Aβ42 and HTTExon1 are all transported in axons but in directions and amounts that are specific of each fibril. After anterograde transport, the three fibrils were secreted in the medium in the absence of axon lysis. Continuous secretion could play an important role in the spread of pathology between neurons but may be amenable to pharmacological intervention.

**Electronic supplementary material:**

The online version of this article (doi:10.1007/s00401-016-1538-0) contains supplementary material, which is available to authorized users.

## Introduction

It is now generally accepted that the prion-like behavior of some aggregation prone proteins is associated with the spread of pathology observed in several neurodegenerative diseases. For Parkinson disease (PD), early evidence came from the startling observation that Lewy bodies, which are in part comprised of α-synuclein (α-syn) fibrils, were present in fetal nigral neurons which had been grafted into the brain of PD patients several years before as a therapeutic measure [24, 26]. This surprising finding suggested that α-syn aggregates may propagate from the host diseased tissue into the graft tissue [10]. The idea of the spread of α-syn pathology within the CNS is strongly supported by a series of recent studies in mice in which stereotaxic delivery of fibrillar α-syn caused the spread of α-syn deposits to anatomically connected brain regions, away from the injection site [28, 29, 36]. Critically, injection of fibrils into α-syn knockout mice caused no pathology, indicating that spread was dependent on templating by endogenous α-syn [28]. Besides α-syn and PD, prion-like spreading may contribute to other neurodegenerative diseases [39]. Similar studies have revealed that proteins associated with Alzheimer disease (AD) and Huntington disease (HD) can also propagate within the nervous system. For AD, there is compelling evidence for the transmissibility of tau pathology in vitro and in vivo [9, 13, 23, 55]. Beta-amyloid, the major component of amyloid plaques in AD, has been shown to propagate in mice even after intraperitoneal injection [15]. For Huntington disease (HD), mutant huntingtin (mHTT) has been observed in neuronal grafts in patients, an observation similar to that made for α-syn in PD, although in this case the deposits were extracellular [8]. Furthermore, mHTT fibrils can spread between neurons in various in vitro culture systems [35, 40].

Early in disease, the lesions of PD, AD and HD are restricted to specific CNS structures. However, with time they spread to more distant, interconnected areas suggesting a role for axonal transport. This is the basis for Braak’s model of PD, which posits that an uncharacterized “agent” spreads in time in a stereotypical manner between areas connected by anterograde or retrograde axonal projections and induces the formation of Lewy bodies [5, 6]. It now appears that the hypothetical “agent” could be misfolded assemblies of α-syn itself. Using primary neurons cultured in microfluidic devices we previously showed that the fibrillar form of α-syn was transported in axons in both anterograde and retrograde directions with kinetics characteristic of the slow component b of axonal transport. Furthermore, after anterograde transport we observed the internalization of the transported fibrils by second order neurons [18], observations congruent with the Braak model. To date, little is known about the neuronal transport of other disease-implicated proteins such as Aβ42 and HTTExon1 under similar experimental conditions. Spread by axonal transport also raises the question of the mechanism of transport and of exit from axons. In particular, is axon degeneration and lysis required for exit, or are fibrils actively secreted by relatively healthy neurons? Such unanswered questions are central to our understanding of the pathogenesis of these neurodegenerative diseases. To address them we devised an assay that measures the amount of fibrils transported under well-defined conditions, in both anterograde and retrograde directions, and we used the assay to compare the axonal transport of α-syn, Aβ42 peptide and HTTExon1. We observed large differences in the transport of the different fibrils. We used two different mouse mutants to determine if lysis was required for their exit from axons. We observed that in all cases exit from axons was independent of lysis, consistent with an active secretion process, which may be amenable to therapeutic modulation.

## Materials and methods

### α-Syn, Aβ42, HTTExon1, Ure2p and Sup35p

The expression and purification of human wild-type α-syn was performed as previously described [19]. α-Syn fibril formation was induced by incubation in 50 mM Tris–HCl, pH 7.5, 150 mM KCl buffer at 37 °C under continuous shaking in an Eppendorf Thermomixer set at 600 r.p.m. The expression and purification of human HTTExon1 with a 48 glutamine stretch was performed as previously described [32]. HTTExon1 was assembled in 20 mM Tris–HCl, pH 7.5, 150 mM KCl, 5 mM MgCl_2_, 1 mM ATP, 100 mM imidazole and 10 % glycerol, at 37 °C without shaking for 24 h. The expression and purification of Met-Aβ 1–42 was performed as described [53]. Aβ 1–42 was assembled in DMEM, at 37 °C without shaking for 24 h.

**Fig. 1:**
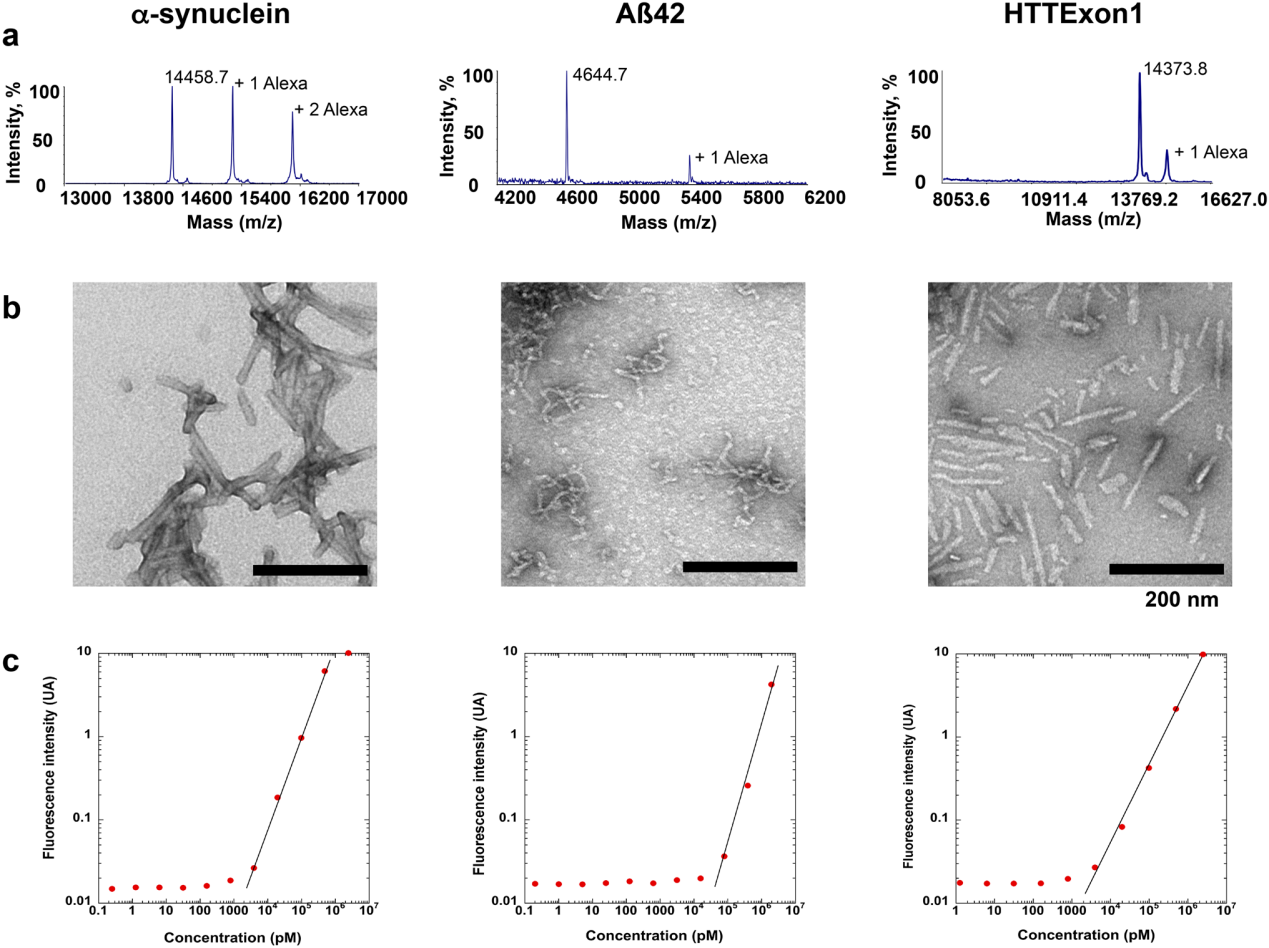
Characterization of α-syn, Aβ42 and HTTExon1 fibrils. **a** MALDI-TOF mass spectra of, from *left* to *right*, Alexa-555 labeled α-synuclein (MH+ 14458.7), Alexa-555 labeled Aβ42 (MH+ 4644.7) and Alexa-555 labeled HTTExon1 (MH + 14,373.8). The number of Alexa 555 molecules covalently bound is indicated. **b** Electron micrographs of the fibrillar forms of α-synuclein, Aβ42 and HTTExon1 used throughout this study. The scale bar represents 200 nm. **c** Calibration curves for Alexa-555 labeled α-synuclein, Alexa-555 labeled Aβ42 and Alexa-555 labeled HTTExon1 within the concentration rang of 1 pM–20 µM

α-Syn, Aβ42 and HTTExon1 fibrils were centrifuged twice at 15,000*g* for 10 min, resuspended twice in PBS. The fibrils were labeled with Alexa-555 (Life Technology, # A-20009) NHS fluorophore following the manufacturer’s instructions using a protein:dye ratio of 1:2. The labeling reactions were arrested by addition of 1 mM Tris pH 7.5. The unreacted fluorophore was removed by a final cycle of two centrifugations at 15,000*g* for 10 min and resuspensions of the pelleted fibrils in PBS. The amount of Alexa-555 incorporated was assessed by mass spectrometry. The samples were de-salted with 5 % acetonitrile, 0.1 % Trifluoroacetic acid (TFA) and eluted from a C18 reversed-phase Zip-Tip (Millipore, Billerica, MA, USA) in 50 % acetonitrile, 0.1 % TFA. Peptide samples were mixed in a ratio of 1:5–1:20 (v⁄v) with sinapinic acid (10 mg/mL) in 50 % acetonitrile and 0.1 % TFA and spotted (0.5 µL) on a stainless steel MALDI target (Opti-TOF; Applied BioSystems). MALDI-TOF-TOF MS spectra were acquired with a MALDI-TOF⁄TOF 5800 mass spectrometer (Applied Biosystems) using linear mode acquisition. External calibration was performed using unmodified α-syn, Aβ42 and HTTExon1. Acquisition and data analysis were performed using the Data Explorer software from Applied Biosystems. The MALDI-TOF mass spectra of Alexa-555-labeled α-syn, Aβ42 and HTTExon1 are shown in Fig. 1a (from left to right). The spectra show that the fractions of labeled α-syn, Aβ42 and HTTExon1 are 60, 20 and 25 %, respectively with, on average, one Alexa-555 per 1, 5 and 4 molecules of α-syn, Aβ42 and HTTExon1, respectively. The nature of the assemblies was assessed using a JEOL 1400 transmission electron microscope following adsorption onto carbon-coated 200-mesh grids and negative staining with 1 % uranyl acetate. The images were recorded with a Gatan Orius CCD camera (Gatan). Representative electron micrographs of fibrillar α-syn, Aβ42 and HTTExon1 are shown in Fig. 1b (from left to right).

Recombinant Ure2p, and hexa-histidine tagged Sup35p were expressed in *E. coli* strain BL21-CodonPlus and purified as described previously [25, 46]. The two yeast prions were assembled into fibrils at 6 °C under gentle agitation (<100 rpm) in 50 mM Tris–HCl, pH 8.0, 200 mM NaCl, 5 % glycerol, 5 mM β-mercaptoethanol, 10 mM MgCl_2_, 2 mM EGTA for Sup35p, 20 mM Tris–HCl pH 7.5, 200 mM KCl, 1 mM EGTA, 1 mM DTT for Ure2p. The assembly reactions were monitored by thioflavin-T binding and transmission electron microscopy observation. The fibrils were labeled with Alexa-555 NHS fluorophore after sedimentation and resuspension in PBS and the unreacted dye was eliminated as described above for α-syn, Aβ42 and HTTExon1 fibrils. Calibration curves (Fig. 1c) were constructed to determine the amount of fibrils by fluorescence spectroscopy using a Quantamaster QM-4/2000 spectrofluorometer equipped with an UXL-75XE short arc lamp (Ushio Inc. Japan). Excitation and emission wavelengths were 550 and 565 nm, respectively. The excitation and emission slits were set at 2 and 6 nm, respectively.

### Primary neuron cultures

C57BL/6 wild type and C57BL/6Sarm1 knock out mutant mice were obtained form Jackson. The C57BL/6Wld^S^ mutant line was a gift from BA Barres (Stanford University Medical School). Primary cortical neurons prepared from E-17 mouse embryos were grown in microfluidic devices (Xona SND 450) for 1 week as described previously [18]. In these devices, anterograde transport can be studied when fluorescent fibrils are added to the soma/dendrite channel and retrograde transport when fibrils are added to the axon channel.

### Fluorescent fibril measurements

To measure the amount of fibrils transported in the anterograde direction, Alexa 555-labeled fibrils were added to the soma channel at a final concentration of 1 µM. The fibril suspension was sonicated for 20 min at 4 °C in a Branson bath sonicator just prior to being added to the culture. The culture was incubated for 24 h. An excess medium volume of 50 µl was maintained in the axon wells and channel during incubation. The medium in the axon wells and channel was harvested after 24 h. The axons were lysed by adding 60 µl of a mixture of detergent (0.1 % SDS, 1 % TritonX100, 0.5 % sodium deoxycholate) to the wells and channel. The lysate was collected and the wells and channel were rinsed once with 60 µl of the detergent mixture. The fluorescence of the medium and lysate was measured with a Quantamaster QM-4/2000 spectrofluorometer (Photon Technology International, Inc., Lawrenceville, NJ) set at 550 nm (excitation) and 565 nm (emission). The amount of fluorescent protein was determined using calibration curves established for each type of pure fibrils for concentrations ranging form 1 pM to 20 µM (Fig. 1c). The same procedure was used to measure the amount of fibrils transported in the retrograde direction except that in this case fibrils were added to the axon wells and channel.

### Western blot analysis

The proteins present in the medium of the microfluidic devices were denatured by incubation at 95 °C for 5 min in 50 mM Tris–HCl, pH 6.8, 4 % SDS, 2 % beta-mercaptoethanol, 12 % glycerol and 0.01 % bromophenol blue, separated in a 10 % polyacrylamide gel and probed with anti α-syn antibody (1:5000, BD Biosciences, 610787). In our hands fibrillar α-syn fully disassembles into monomers in sample buffer and runs as a single band with a molecular weight of 14 kDa [36].

### Internalization of fibrils

The technique was described previously [43]. Briefly, primary cultures of neurons (5 × 10^4^ neurons per well) were prepared in 8 well LabTek chamber slides (Nunc). After 1 week of culture, Alexa488 labeled fibrils were added at a final concentration of 1 µM. Internalization was measured at 1, 3, 5, 7 h after the addition of fibrils. At each time point, the medium was removed, the cells were washed 3 times with PBS. Trypan blue (0.1 % in PBS) was added to each well and the cells were observed using a phase contrast/fluorescence microscopic set up with a 20× objective. Pictures of 15–20 tiled frames were taken and stored for further analysis. Control wells without fibrils were used to ensure the absence of background fluorescence. The percentage of neuron containing fluorescent puncta was determined manually by counting more than 100 neurons for each well.

### Immunostaining

Cells were fixed with 4 % paraformaldehyde in PBS for 30 min at room temperature, followed by 4 washes with PBS. For SMI-31 and SMI-32 immunostaining, antigen retrieval was performed with the Retrieve-All3 Buffer (Covance, SIG-31904-500) at 95 °C for 10 min, followed by 4 washes with PBS. Incubation with the primary SMI-31 and SMI-32 antibodies (1/1000) was done in 3 % BSA, 0.1 % TritonX100 in PBS, overnight at 4 °C. Following washes in PBS the secondary antibody (a goat anti-mouse *Ig* coupled to Alexa 488 fluorochrome (ThermoFisher)) was incubated for 1 h at room temperature, using the same buffer. For the EEA1 (CST, 3288), Rab5 (CST, 3547), Rab7 (CST, 9367), Lamp1 (DSHB, 1D4B), BIII-tubulin (Synaptic systems, 303 304), MAP2 (Synaptic systems 188 004) and a-syn (Abcam, ab138501) immunostainings, primary antibodies were diluted in 5 % goat serum, 0.3 % TritonX100 in PBS and incubated on fixed cells overnight at 4 °C. Alexa Dye-conjugated goat anti-rat, -rabbit and -guinea pig antibodies were used as secondary antibodies (ThermoFisher).

### Reagents

The microfluidic devices were from Xona (SND 450). SMI-31, SMI-32 monoclonal antibodies were from Covance. EEA1, Rab5, Rab7 monoclonal antibodies were purchased from Cell signaling technology (CST), Lamp1 from DSCB, BIII-tubulin and MAP2 from Synaptic systems. Alexa Dye-conjugated goat secondary antibodies were from ThermoFisher.

## Results

### Comparing anterograde and retrograde transport of α-syn, A-β42 and HTTExon1 fibrils

**Fig. 2:**
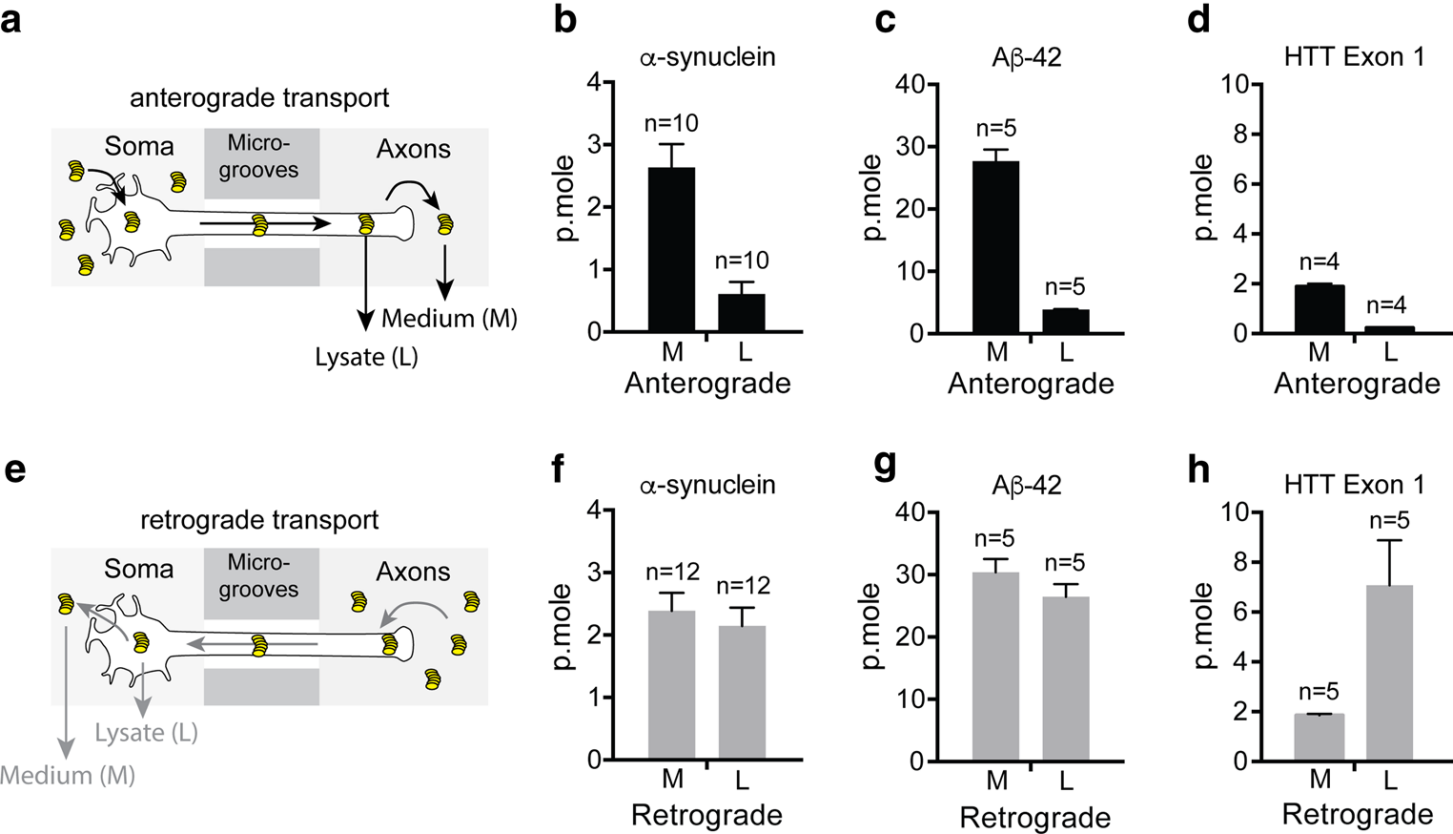
Axonal transport of fibrils in microfluidic devices. Quantity (p.moles) of fibrillar α-syn, Aβ42 and HTTExon1 transported by axons in 24 h in the anterograde and retrograde directions. Primary embryonic cortical neurons were grown in microfluidic devices. Fibrils (1 µM monomer) were added to the soma or axon compartments. Fluorescence was measured in the opposite compartment after 24 h of incubation. **a**–**d** Anterograde transport. **e**–**h** Retrograde transport. **a**, **e** Schematics of the experimental design. Fibrils are depicted as coin piles. **b**–**h** Ordinates give the number of picomoles of fibrils transported per microfluidic device in 24 h. Note that the ordinate values are different for the three kinds of fibrils but that, for a given fibril, they are the same for anterograde and retrograde transport. *M* fibrils spontaneously released in the medium. *L* fibrils released after lysis by detergents of the axons or soma for, respectively the anterograde and the retrograde transport. *n* number of microfluidic devices used for the measurement

Microfluidic devices, which physically separate neuron cell bodies from their axons, are ideally suited to study both the anterograde and retrograde transport of fibrils by axons [47]. We previously used this technique to characterize the transport of α-syn fibrils in axons of primary embryonic mouse neurons [18]. In the present work we extended these observations to fibrillar forms of the Aβ42 peptide and HTTExon1 to determine if these distinct protein assemblies would be transported by similar or different mechanisms (Fig. 1). For studies of anterograde transport, we added fluorescently labeled fibrils to the channels with neuronal soma and measured the fluorescence released in the axon channels after 24 h (Fig. 2a–d). To measure retrograde transport, we added the labeled fibrils to the axon channels and measured the amount transported to the soma channels (Fig. 2e–h). For both anterograde and retrograde transport, we measured the fluorescence of the medium as well as the fluorescence released after lysis of cellular structures with detergents. Fluorescence of medium represented proteins spontaneously released in the medium after transport. Fluorescence of the lysate represented fibrils present in axons or soma/dendrites after anterograde and retrograde transport, respectively. We used calibration curves (see “Materials and methods”, Fig. 1c) to determine the number of picomoles of monomeric proteins transported from the fluorescence values. We used fluorescent cholera toxin subunit B (CTB) to estimate the number of axons per microfluidic device (Supp. Figure 2, Table 1). When added to axon termini, CTB binds to plasma membranes, is internalized and diffuses into the entire neuron arborescence (Supp. Figure 2). Part of it migrates to the perinuclear region of the Golgi cisternae [3, 17]. Since CTB is not released from the cells in which it has been internalized, only soma whose axons entered the microgrooves are labeled (Table 1). In Table 2, the results of transport of each fibril are displayed as the number of femtomoles of monomeric protein transported per axon in 24 h.

**Table 1:** Number of axons in microfluidic devices

Experiment nb.	CTB^ +^ soma	α-Syn^ +^ soma	% axons transporting α-syn
1	290	166	57 %
2	253	133	53 %
3	294	170	58 %
4	190	n.d.	–
5	258	n.d.	–
6	178	n.d.	–
7	281	n.d.	–
8	235	n.d.	–
9	242	149	62 %
10	278	64	23 %

Alexa 488-labeled CTB (5 ng/µl) was added to the axon channel of 7 day-old neuron cultures. After 24 h the cultures were fixed with 4 % paraformaldehyde. The soma channel was scanned with a fluorescence microscope equipped with a ×40 lens. The CTB positive soma were counted. In some experiments, Alexa 555-labeled α-syn fibrils (10 µM) were mixed with CTB and the number of CTB and α-syn positive soma was determined. *n.d.* No fibrils were added in these experiments

**Table 2:** Amount of fibrils (femtomoles) transported in the anterograde and retrograde direction in 24 h

Sample	α-Syn	A-β 42	HTT Exon1
Anterograde, medium	10.4	111.2	7.6
Anterograde, lysate	2.4	16.0	0.8
Anterograde, total	12.8	127.2	8.4
Retrograde, medium	9.6	120.8	7.6
Retrograde, lysate	8.4	106.4	28.4
Retrograde, total	18.0	227.2	36.0
Antero./retro.	0.71	0.56	0.23
% released in medium after anterograde transport	81 %	87 %	90 %

Consistent with our previous findings, α-syn, was transported in both anterograde and retrograde directions (Fig. 2b, f, Supp. Figure 1b, e, Table 2). Aβ42 peptides were also transported in both directions but strikingly the amount of protein that appeared in axons after anterograde transport and in soma/dendrites after retrograde transport was more than ten times higher than that observed with α-syn fibrils (Fig. 2c, g, Supp. Figure 1c, f, Table 2). HTTExon1 fibrils were not significantly transported in the anterograde direction but retrograde transport proceeded similarly to that of α-syn fibrils (Fig. 2d, h, Supp. Figure 1g, Table 2). The ratio of anterograde versus retrograde transport was lower for HTTExon1 fibrils than for the other two (0.2 for HTTExon1 fibrils versus 0.7 and 0.6 for α-syn and Aβ42 fibrils, respectively). Interestingly the majority of HTTExon1 fibrils remain intracellular after retrograde transport, whereas approximately 50 % of α-syn and Aβ42 fibrils were released into the medium.

After retrograde transport, the signal given by fluorescently labeled α-syn overlapped that obtained by immunostaining with an anti-α-syn antibody (Supp. Figure 3a, b), suggesting that the internalized and transported α-syn was not degraded during the course of these experiments. Additionally, we observed that part of the internalized and retrogradely transported fluorescently labeled fibrils overlapped with late endosomal and lysosomal markers, in particular Rab7 and Lamp1 but not early endosomal marker EEA1 or Rab5 (Supp. Figure 4 and Supp. Figure 5). Furthermore, the majority of fibrils transported in the anterograde direction (85 %) were released from the axons into the medium (Table 2) and the released α-syn was intact, un-degraded protein (Supp. Figure 3c).

**Fig. 3:**
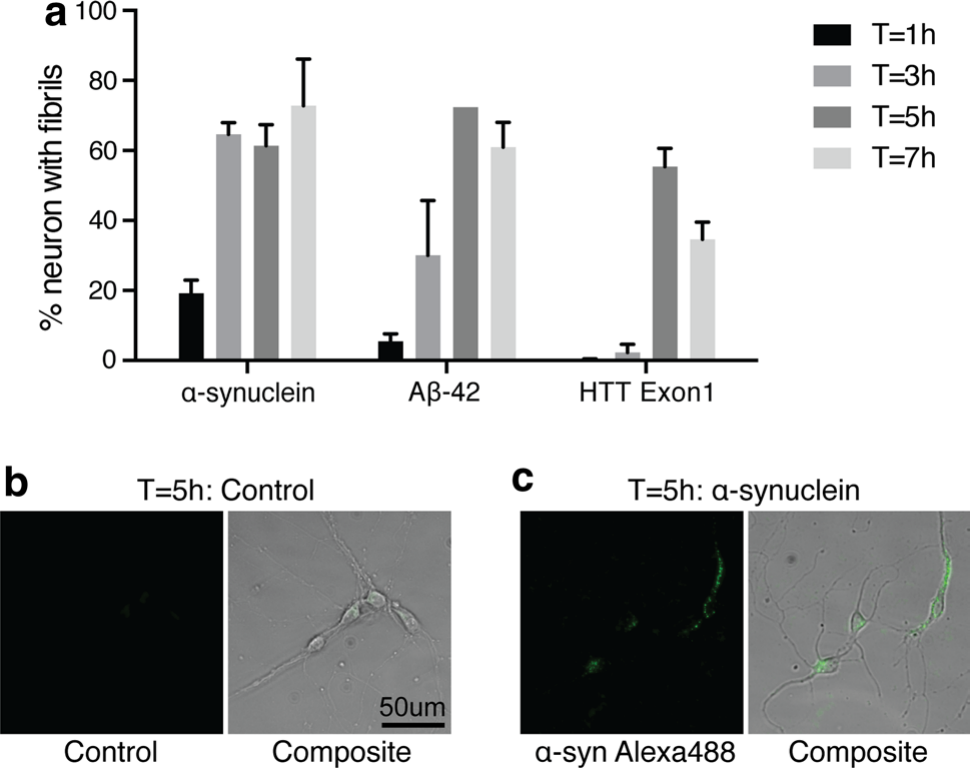
Internalization of fibrils by neuron soma. Primary embryonic cortical neurons were grown in 8 well LabTek slides at low density. Alexa488-labeled fibrils (1 µM monomer) were added and incubated for 1, 3, 5, and 7 h. At each time point the fibrils were removed; the cells were washed with PBS; 0.1 % Trypan Blue in PBS was added and pictures (phase contrast and fluorescence) of tiled fields of the cultures were recorded. **a** Quantification of the  % of neurons that internalized fibrils was determined for a minimum of 150 neurons. Four independent experiments were performed. **b**, **c** Representative bright field and fluorescent images of primary cortical neurons 5 h after exposure to α-syn fibrils or control medium

Since internalization, the first step in the transport of fibrils, could be rate limiting, we compared the rate of fibril internalization by primary cortical neurons. We measured the fraction of neurons that internalized fibrils as a function of time of incubation (Fig. 3a–c). The values were similar for α-syn, Aβ42 and HTTExon1 and we observed that by 5 h approximately 60 % of all neurons had internalized fibrils added to the culture medium. The number of fluorophores per molecule and the molecular weight of the protein (which determines the number of subunit per fibril of a given size) are comparable for Aβ42 and HTT40 (see “Materials and methods”). Therefore, Fig. 3 suggests that the number of fibrils internalized as a function of time is similar for both. Nonetheless, the amount of fibrils transported and released in the medium is considerably higher for Aβ42.

### The transport of fibrillar forms of yeast prions Sup35p and Ure2p

**Fig. 4:**
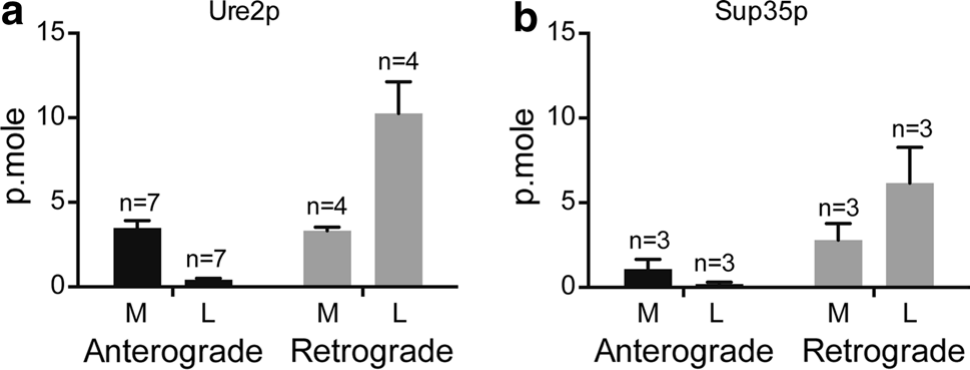
Transport of the fibrillar forms of Ure2p and Sup35p yeast prions by mouse neurons. The experiment was performed as for α-syn, Aβ42 and HTTExon1 fibrils (Fig. 2). Ure2p fibrils were obtained as described [49]. Sup35p fibrils were obtained as described [25]

Our results provide evidence that the transport of fibrillar proteins by neurons depends greatly on the nature of the protein. To explore this further, we next analyzed transport of fibrillar forms of 2 yeast prions, Sup35p and Ure2p, which both form fibrils that can be internalized by mammalian cells. Both yeast prions were transported in amounts and patterns (anterograde versus retrograde transport) similar to HTTExon1 fibrils and different from those of α-syn and Aβ42 fibrils (Fig. 4a, b). These data provide further evidence that axonal transport is strongly dependent on the nature of the fibrillar protein. It also indicates that the transport of fibrils by neurons does not require the presence of an endogenous homologous protein.

### The release of fibrils from axons

**Fig. 5:**
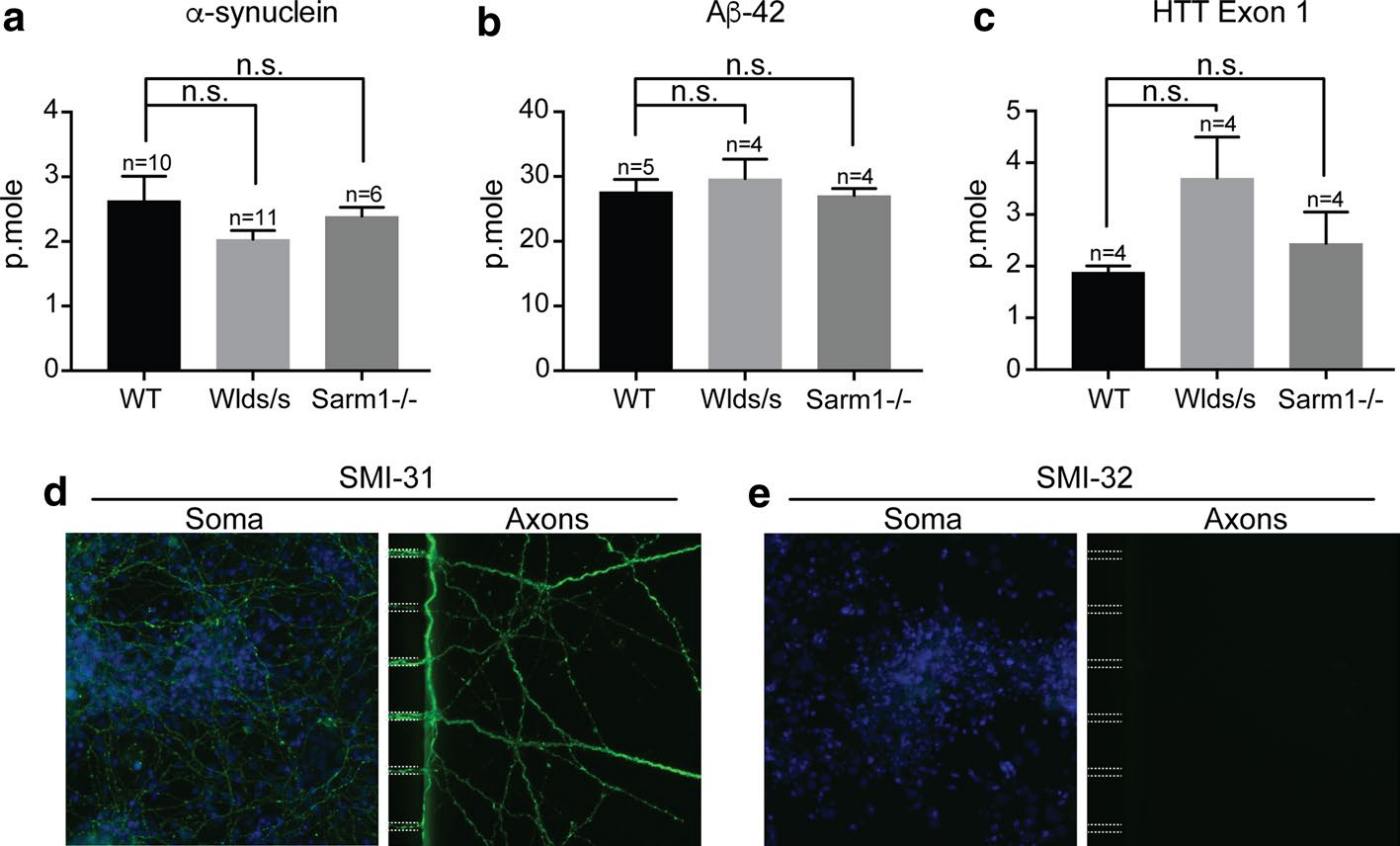
Quantity of α-syn, Aβ42 and HTTExon1 released in the medium in 24 h of anterograde transport by axons from C57BL/6wt (WT), C57BL/6Wld^S^ (Wlds/s) and C57BL/6Sarm1^−/−^ (Sarm1^−/−^) mice. Primary embryonic cortical neurons from mice of the 3 genotypes were grown in microfluidic devices. Fibrils were added to the soma compartments. The fluorescence of the medium in the axon compartments was measured after 24 h of incubation. *n* number of microfluidic devices used for the measurement. *n.s* Not statistically significant. **d**, **e** Staining with the SMI-31 and SMI-32 monoclonal antibodies of the soma and axon of Wld^S^ neurons in microfluidic devices after 24 h of transport of α-syn fibrils. Similar results were obtained with WT and Sarm1^−/−^ neurons and with Aβ42 and HTTExon1 fibrils. SMI-32 did not detect un-phosphorylated neurofilaments, a sign of axonal damage. A positive control for the SMI-32 antibody was obtained by treating the soma with staurosporin to induce apoptosis (not shown)

It has been proposed that fibril toxicity is responsible for neuron death in neurodegenerative diseases [37, 52]. Therefore, we wondered whether the release of the proteins under study in the medium after anterograde transport could be the result of neuronal injury and axon lysis. To test this hypothesis, we performed experiments with neurons whose axons are intrinsically resistant to degeneration. We used neurons from two mouse mutants, the spontaneous Wld^S^ mutant and the Sarm1 knockout. Axons from these mutants are remarkably resistant to injury, remaining structurally and functionally intact in the mouse, weeks after nerve transection and in culture weeks after axotomy [11, 31, 34, 54]. We reasoned that if the proteins were released from axons owing to axon lysis, the Wld^S^ and Sarm1^−/−^ mutations should reduce the amount of released fibrils. If, on the other hand, fibrils were released by an active process, independent of axon lysis, the amount of fibrils released from these mutant neurons should not differ from the amount released by WT neurons. We isolated neurons from WT, Wld^S^ and Sarm1^−/−^ mice, all with the same C57Bl/6 background. We first confirmed that mutant axons remained intact after 24 h of incubation with fibrils, using SMI-31 and SMI-32 monoclonal antibodies, which are specific for phosphorylated and unphosphorylated neurofilaments, respectively (Fig. 5e, f). Next, we measured the release of α-syn, Aβ42 or Htt exon 1 proteins in the medium following anterograde axonal transport. For the three proteins, the amount of protein released was not statistically different for WT and mutant neurons (Fig. 5a–c). This result indicates the three different fibrils were secreted by axons in the absence of axonal lysis.

## Discussion

The work of Braak and his collaborators indicates that the lesions of PD and AD spread in time along both anterograde and retrograde axonal projections [5, 6, 48]. The ability of fibrillar α-syn to be internalized by neurons and to promote the fibrillization of endogenous α-syn, has been documented [4, 12, 21, 30, 33]. Together these results form the basis for a model in which the prion-like properties of α-syn fibrils and their axonal transport might be responsible for the progression of PD pathology. In a previous publication, we reconstructed two steps of this model using primary cortical mouse neurons grown in microfluidic devices. We showed that the α-syn fibrils were transported in axons in both the anterograde and the retrograde direction and that, after anterograde transport some of them were internalized by a second order neuron [18]. In the present work we quantified the axonal transport of fluorescently labeled α-syn fibrils by measuring the fluorescence delivered to the receiving compartment of the microfluidic device. Using calibration curves that relate fluorescence to concentration and using CTB to determine the number of axons, we measured the number of α-syn molecules transported in 24 h per axon in both the anterograde and retrograde direction (Fig. 2b, f; Table 2). On average an axon transported 12.5 femtomoles of α-syn in 24 h in the anterograde direction. Interestingly, in the case of retrograde transport, we determined that only about 55 % of the axons transported fibrils (Table 1). As a result, each of these axons transported approximately 40 femtomoles of α-syn in 24 h. Since primary cultures of cortical neurons are a heterogeneous population, the fact that only half of them transported fibrils may indicate the existence of specificities according to the origin of the neuron, which, if true, could have relevance to pathogenesis.

We compared the transport of fibrils of α-syn, Aβ42 and HTTExon1, and observed large differences. The most striking one was with Aβ42 fibrils, which were transported in approximately ten times higher amounts than α-syn and HTTExon1 fibrils. The large amount of fibrils that accumulate in the soma after retrograde transport raises the issue of their intra-cytoplasmic localization. Using 3-color immunofluorescence and confocal microscopy, we showed that most of of them co-localize with the endosome/lysosome markers Rab7 and Lamp1. This holds true for α-syn Aβ42 and HHTExon1 (Supp. Figure 3). It may indicate that the endosome/lysosome pathway is involved in the secretion of these proteins after their retrograde transport. Interestingly, there is good evidence that although Aβ42 oligomers interfere with fast axonal transport of organelles [14, 22, 46] fibrillar Aβ42 does not [38], an observation that is consistent with our results. Also, evidence is mounting that the intraneuronal accumulation of fibrillar Aβ42 could play an important role in AD pathology [20, 45]. Therefore, the efficient axonal transport of Aβ42 fibrils and their accumulation in soma after retrograde transport could be important factors in the pathogenesis of AD. The ratio of anterograde to retrograde transport also varied widely between the fibrils, with HTTExon1 fibrils being transported mainly in the retrograde direction. Overall, our results show that steps such as internalization, cytoplasmic addressing to the axon or transport per se, are performed by neurons with very different efficiencies depending on the nature of the fibrils. We examined the part that internalization may play in these differences. As shown in Fig. 3a, Aβ42 and HTTExon1 were internalized at similar rates although they were transported with very different yields. A combination of physico-chemical properties of fibrils, such as size, stiffness, surface charge and hydrophobicity may determine steps in axonal transport subsequent to internalization. Fibrils comprised of the same protein can adopt distinct conformations which may contribute to distinct clinical features [1, 27, 36]. These strain specific conformations of fibrils could alter their axonal transport.

We showed previously that the kinetics of anterograde axonal transport of α-syn fibrils corresponds to that of slow component b [18]. Slow component b transports a large variety of axonal proteins, including α-syn, from their cytoplasmic site of synthesis to their delivery sites along the axon. These proteins are transported in large assemblies, which are not enclosed in a membrane [41, 42, 44]. The molecular link that attaches the assemblies to the kinesin motors has not been identified. We hypothesize that fibrillar α-syn, Aβ42 and HTTExon1 may share surface properties, and possibly size, with slow component b protein assemblies, that could serve to introduce them in this pathway of axonal transport.

An important aspect of the present work is the demonstration that the proteins under study were secreted from axons after anterograde transport (Fig. 2a–d). This is consistent with recently published results obtained in a mouse model [51]. Secretion of α-syn, including its fibrillar form, in exosomes has been suggested by the work of others [2, 7, 16, 50]. Exosomes may fuse with the plasma membrane of a target neuron, delivering fibrils in the cytoplasm where it could seed the misfolding of endogenous α-syn. Continuous secretion of α-syn fibrils by axons may contribute to the spread of lesions in the CNS of PD patients. It is now important to understand the molecular details of the transfer of fibrils from axons to a second order neuron to devise specific therapies to halt the progression of neurodegenerative diseases such as PD.

## Electronic supplementary material

Supplementary material 1 (TIFF 12813 kb)

Supplementary material 2 (TIFF 5326 kb)

Supplementary material 3 (TIFF 11655 kb)

Supplementary material 4 (TIFF 33479 kb)

Supplementary material 5 (TIFF 33479 kb)
