# Supplementary figures and images for: Axonal transport and secretion of fibrillar forms of α-synuclein, Aβ42 peptide and HTTExon 1
Source: Acta Neuropathol. 2016 Jan 28;131:539–48. doi: 10.1007/s00401-016-1538-0 (PMC4789229; doi:10.1007/s00401-016-1538-0)

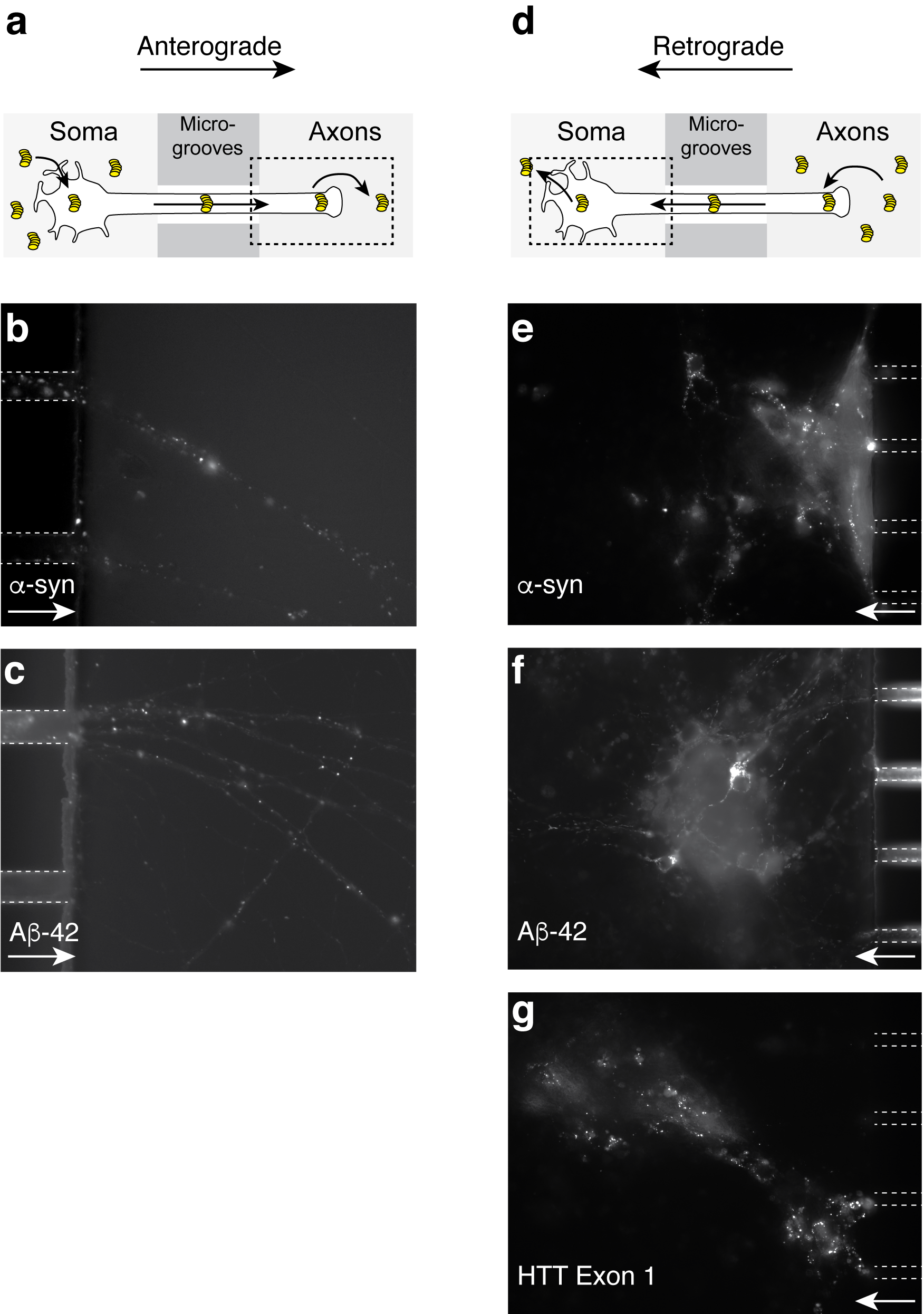

Supplement: Supplementary file 1 — Supplementary material 1 (TIFF 12813 kb) [file 401_2016_1538_MOESM1_ESM.tif]

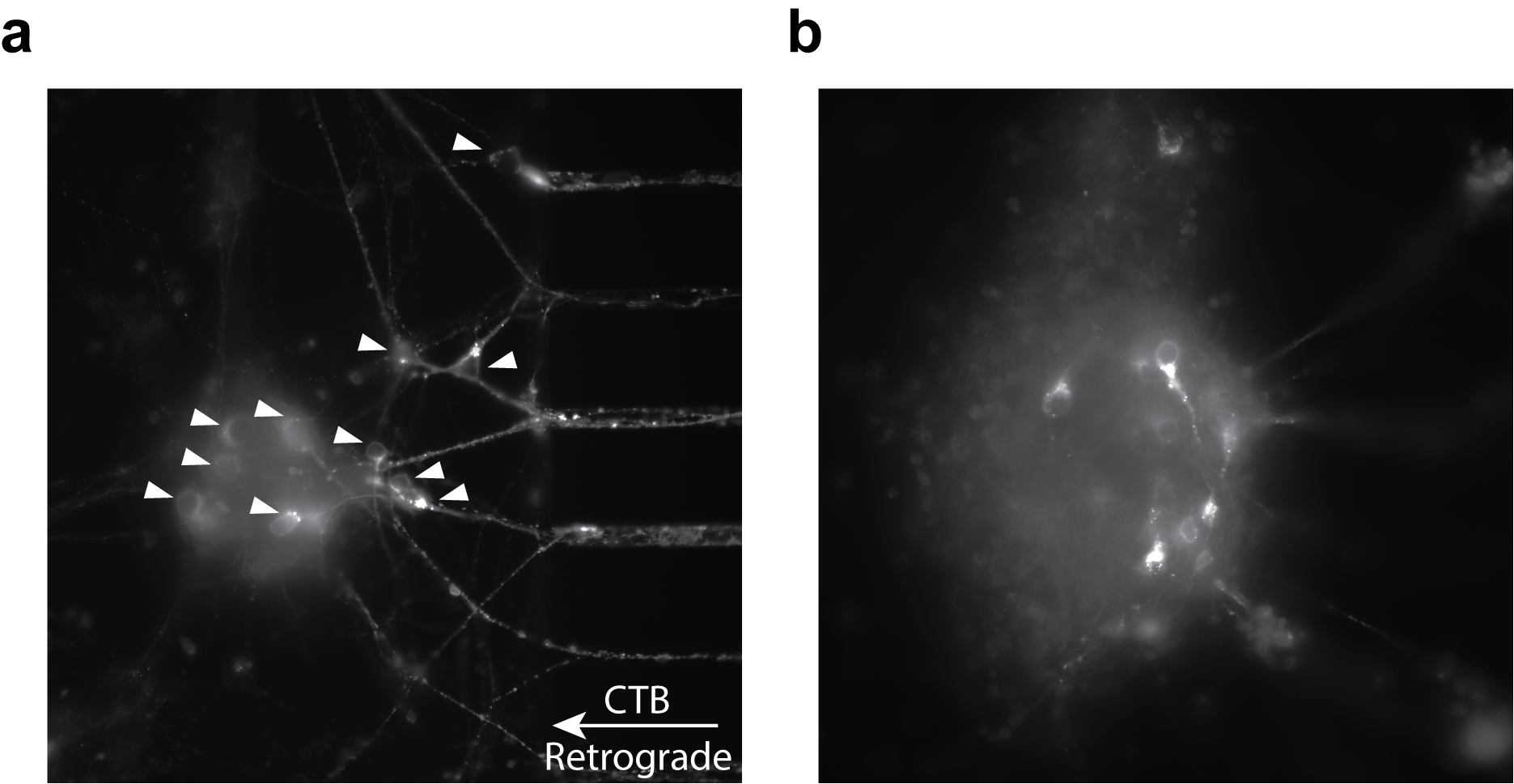

Supplement: Supplementary file 2 — Supplementary material 2 (TIFF 5326 kb) [file 401_2016_1538_MOESM2_ESM.tif]

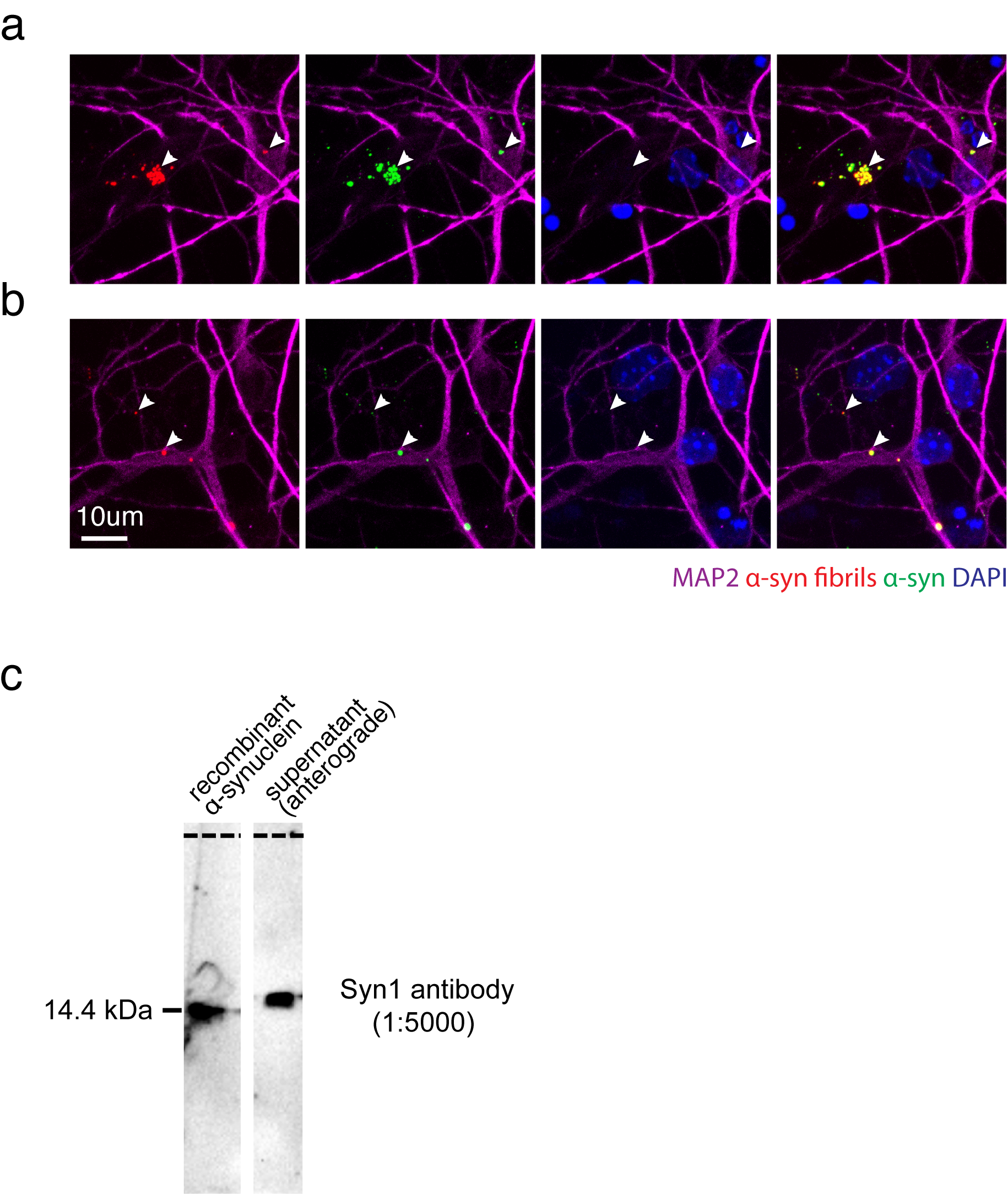

Supplement: Supplementary file 3 — Supplementary material 3 (TIFF 11655 kb) [file 401_2016_1538_MOESM3_ESM.tif]

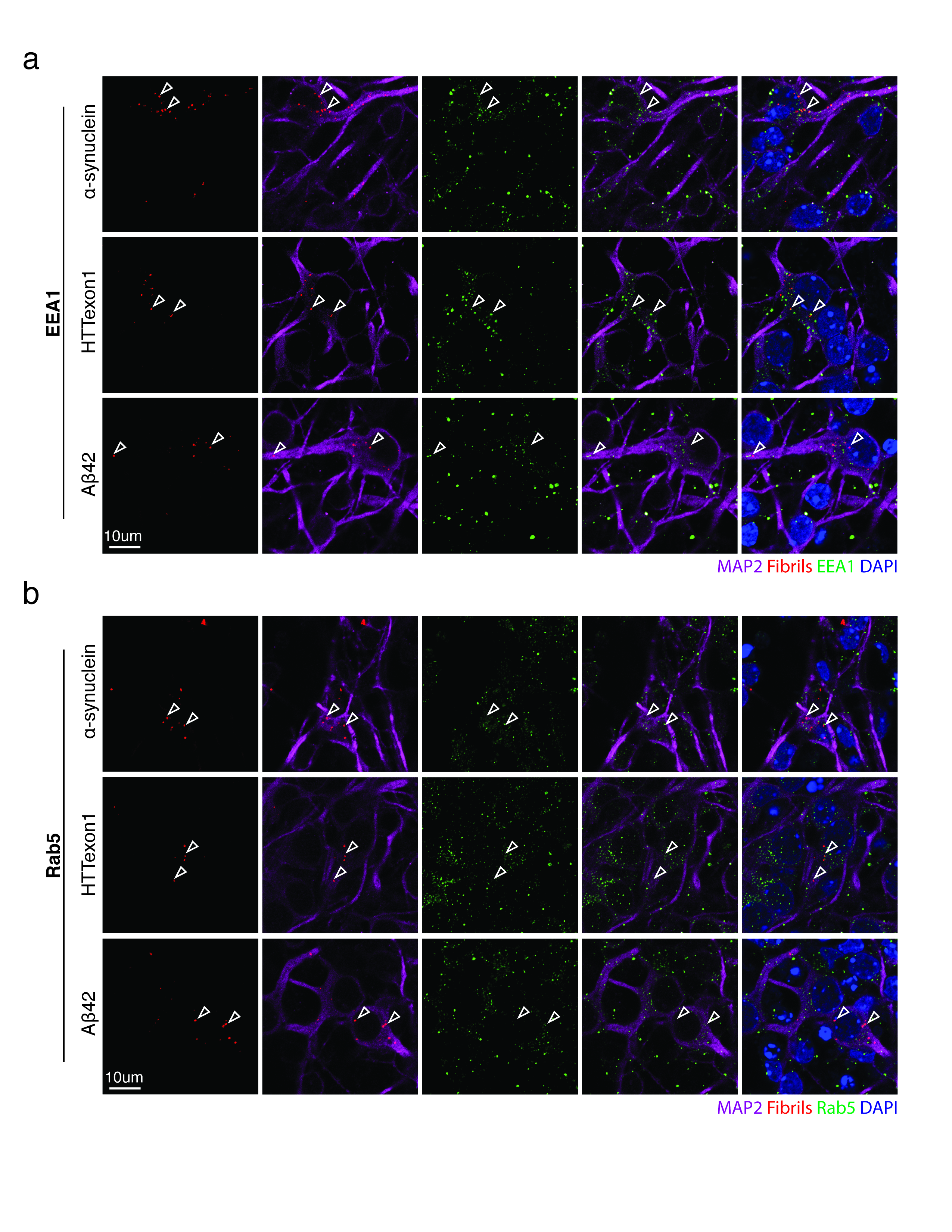

Supplement: Supplementary file 4 — Supplementary material 4 (TIFF 33479 kb) [file 401_2016_1538_MOESM4_ESM.tif]

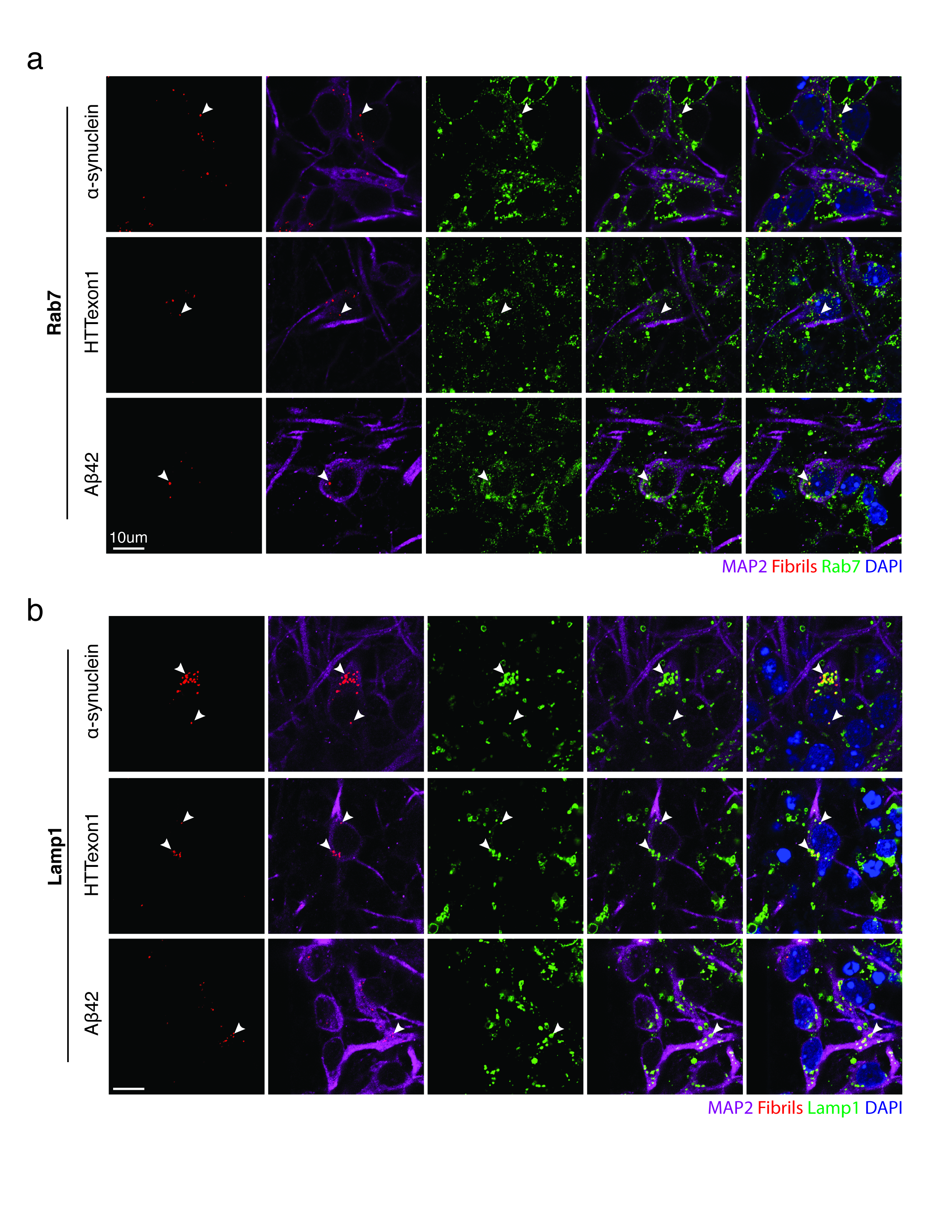

Supplement: Supplementary file 5 — Supplementary material 5 (TIFF 33479 kb) [file 401_2016_1538_MOESM5_ESM.tif]
